# Dynamic response mechanism of the hole subjected to the 3D point source disturbance in an isotropic formation

**DOI:** 10.1038/s41598-024-67417-8

**Published:** 2024-07-19

**Authors:** Wang Hongwei, Du Wei

**Affiliations:** 1 Faculty of Civil Engineering and Mechanics, Kunming University of Science and Technology, Kunming, China; 2 School of Earth Sciences, Yunnan University, Kunming, China

**Keywords:** Helmholtz potentials, Sommerfeld integral, Wave propagation, Civil engineering, Mechanical engineering, Solid Earth sciences, Geophysics

## Abstract

Accurate estimation of the effects of dynamic disturbances on stress concentration is crucial for the stability of rock engineering and the corresponding analytical approaches are needed. This study presents an analytical approach to calculate the relative stress distribution with a point source inside and outside a hole using the linear theory of elasticity. The Helmholtz potentials and Sommerfeld integral are employed to describe the displacement and stress components, and then formulate the equilibrium equations to solve the equivalent stress distribution around the hole. Numerical examples demonstrate the impact of model parameters on the equivalent stress, such as frequency, hole radius, source location, etc. It is found that sometimes high frequencies can make the equivalent stress greater far from the source than that close to the source. Additionally, when the ratio of the distance between the source and the hole axis to the hole radius exceeds ten, the equivalent stress distribution around the hole remains nearly constant. This approach can be used for the design and assessment of underground engineering structures' stability under dynamic disturbances.

## Introduction

Understanding the dynamic response under 3D point source disturbances provides critical insights into the behavior of geological formations^1^, such as extensive efforts have been dedicated to both theoretical and empirical studies on the spatiotemporal evolution of fractured rock masses^2–4^. Most dynamic responses are primarily governed by mechanical parameters, geometric shape, boundary conditions, etc.^5^. For example, researchers often prefer body waves for analyzing structural defects^6^, artificial activities^7^, underground physical properties^8–10^, and even the initiation of cracks^11–13^. Here we are focusing on dynamic response mechanism of holes subjected to the 3D point source disturbance in isotropic formations and solving the corresponding analytical approach.

To solve the above problem, the first step is to accurately evaluate the mechanical properties for rocks surrounding the hole. In recent years, various equivalent medium models have been proposed to simplify the dynamic response of the hole models, such as the isotropic model^8,14^, VTI model^15–17^, HTI model^17^, TTI model^10,18^, and mono/orth model^19^. With increasing frequency of external forces, the properties shift from elasticity to viscoelasticity, as seen in the works of Gist^20^, Chapman^21^, Biot^22,23^, Schoenberger and Levin^24^, and Johnston et al.^25^. However, the equivalent models primarily consider the overall effect of the fractures rather than the interactions between individual or multiple sets of fractures. For example, Fang et al.^26^ simulated the response of the non-uniform distribution of fractures and point out the differences from the enforced use of the equivalent theory.

In terms of geometry influencing, such as the interconversion between spherical waves, cylindrical waves, and plane waves, the general solutions to related problems can be accurately obtained through boundary or interface shapes^7,8^. Even scholars derive the methodology for computing the stresses around an arbitrarily-shaped hole^27^. On the other hand, if mathematical expressions are not available, finite element methods (FEM)^28^ are used as an alternative, but these methods may not accurately interpret the effects of dynamic disturbance. For example, Morse^29^ and Buchwald^30^ studied the propagation of body waves in a hole in homogeneous transversely isotropic media, while Fang et al.^31^ used FEM to analyze the propagation around the hole in inhomogeneous media. Additionally, the existing research has some limitations, such as assume the body waves to be plane waves, ignore the spatial position of dynamic disturbance^32,33^, focus on the wave propagation around the hole and not develop the reasonable method to predict the attenuation of dynamic disturbances caused by the hole^34,35^.

In this study, we assume the hole to be embedded in an isotropic formation and then derive the analytical approach to calculate the stress distribution caused by the 3D point source. Before performing the calculation, we solved general displacement equations taking into account the source position^36,37^. Based on different boundary conditions, we solved for the stress distribution to gain a deeper understanding of how to effectively protect buried infrastructures from dynamic disturbance, in a word, the proposed model provides a valuable tool for the design and assessment of structures in dynamic disturbance regions.

## Problem description

**Figure 1:**
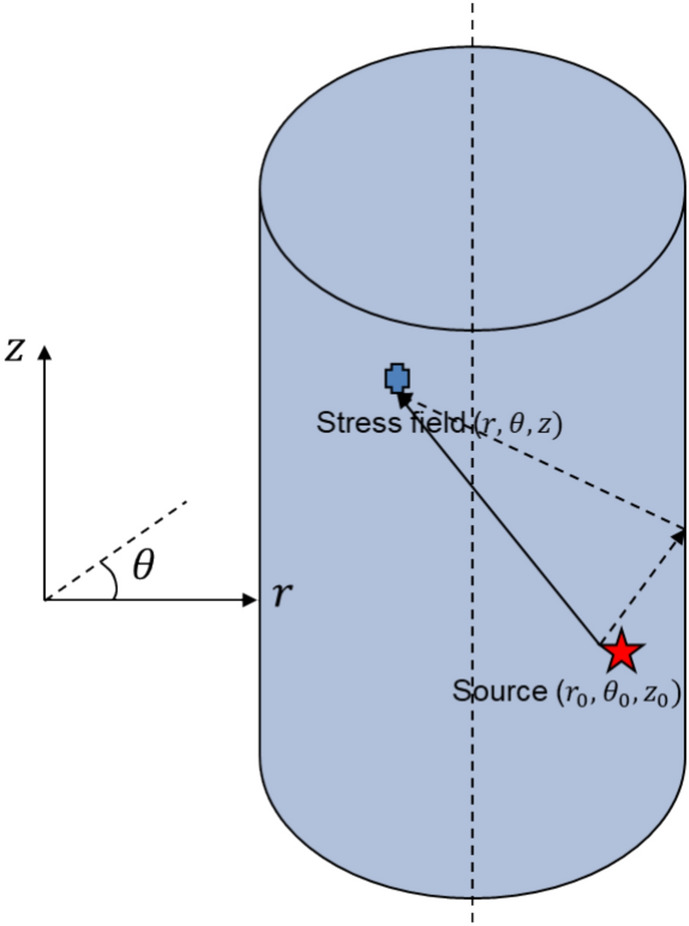
Locations of point source and observing stress point in the cylindrical system.

Drilling a hole in an infinite isotropic medium, the point source could be located inside or outside the hole. The stress wave emitted by the point source generates reflections and transmissions at the continuous solid–liquid interface, e.g. $({r}_{0},{\theta }_{0},{z}_{0})$ in Fig. 1, and the corresponding boundary conditions are expressed as

1 $${u}_{r}^{(1)}={u}_{r}^{(2)},{\sigma }_{rr}^{(1)}={\sigma }_{rr}^{(2)},{\sigma }_{r\theta }^{(2)}=0,{\sigma }_{rz}^{(2)}=0$$

where superscript 1 indicates liquid, 2 indicates solid, ${u}_{j}^{(i)}$ for displacement and ${\sigma }_{j}^{(i)}$ for stress. Without liquid in the hole, the boundary conditions are becoming (free boundary)

2 $${\sigma }_{rr}^{(2)}=0,{\sigma }_{r\theta }^{(2)}=0,{\sigma }_{rz}^{(2)}=0$$

The following sections will provide a detailed derivation of the analytical solution for stress distribution on Eq. (1). About the analytical solution on Eq. (2), only the final result will be presented, whose solving process is almost the same to that of Eq. (1).

## Waves in an isotropic medium

The wave equation under the action of Dirichlet external forces are expressed as follows^33^:

3 $$\left({\nabla }^{2}-\frac{\rho }{\lambda +2\mu }\frac{{\partial }^{2}}{\partial {t}^{2}}\right)\phi =\delta \left(r-{r}_{0}\right)\delta \left(\theta -{\theta }_{0}\right)\delta \left(z-{z}_{0}\right)f(t)$$

where $\phi$ is the displacement potential, $f(t)$ is the source time function, $\delta$ is a Dirichlet function $({r}_{0},{\theta }_{0},{z}_{0})$ are the source coordinates along radial $r$, azimuthal $\theta$ and axial $z$ directions,$\rho$ the density,$\lambda$ and $\mu$ the lambda constants. The general solution for $\phi$ in Eq. (3) can be expressed as^8^

4 $$\phi =\sum_{n=0}^{\infty }\left\{{\varepsilon }_{n}{K}_{n}\left({k}_{r}{r}_{>}\right){I}_{n}\left({k}_{r}{r}_{<}\right)\right\}\text{cos}\left[n\left(\theta -{\theta }_{0}\right)\right]*f(t){e}^{i{k}_{z}(z-{z}_{0})}$$

with

5 $${r}_{>}=\text{max}\left(r,{r}_{0}\right),{r}_{<}=\text{min}(r,{r}_{0})$$

6 $${k}_{r}=\sqrt{{k}_{p}^{2}-{k}_{z}^{2}}$$

7 $${k}_{p}=\frac{\omega }{{v}_{p}}=\omega \sqrt{\frac{\rho }{\lambda +2\mu }}$$

where ${\varepsilon }_{n}=\frac{1}{\pi }$ when $n=0$, ${\varepsilon }_{n}=\frac{2}{\pi }$ for other cases, ${I}_{n}$ and ${K}_{n}$ is the first and second types of the modified Bessel function, respectively, ${k}_{r}$ is the complex wavenumber in the radial direction, and $\omega$ is the angular frequency. Place the source in the liquid medium and set $(\lambda ,\mu , \rho )$ to be $({\lambda }_{1},0,{ \rho }_{1})$ while $({\lambda }_{2},{\mu }_{2},{ \rho }_{2})$ in the solid medium.

The general solution for the time function $f(t)$ is ${e}^{iwt}$. To simplify the form of the equations, the subsequent expressions will omit the time function term. So the expression for P-wave propagating close to the hole axis at the solid–liquid interface is as follows:

8 $${\phi }_{1}\left(r,\theta \right)=\sum_{n=0}^{\infty }{D}_{n}{I}_{n}\left({k}_{1p}r\right)\text{cos}\left[n\left(\theta -{\theta }_{0}\right)\right]*{e}^{i{k}_{z1p}(z-{z}_{0})}$$

**Figure 2:**
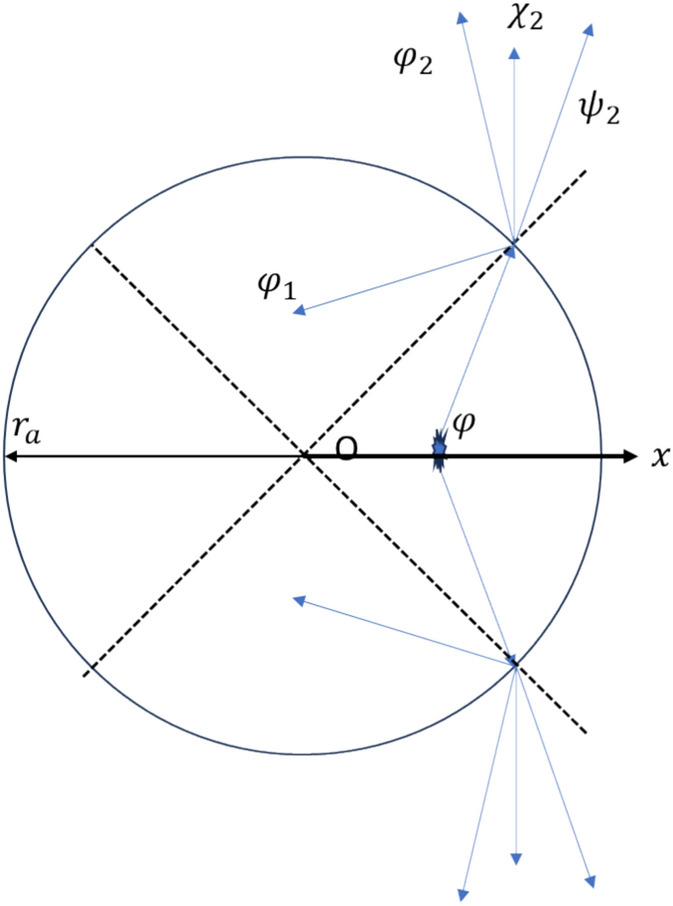
The cross-sectional plane of the hole model with hole radius *r*_*a*_.

The expressions for the P-, SH- and SV-waves propagating away from the hole axis are (Appendix A),

9 $${\phi }_{2}\left(r,\theta ,z\right)=\sum_{n=0}^{\infty }{A}_{n}{K}_{n}({k}_{2p}r)\text{cos}[n(\theta -{\theta }_{0})]*{e}^{i{k}_{z2p}(z-{z}_{0})}$$

10 $${\chi }_{2}\left(r,\theta ,z\right)=\sum_{n=0}^{\infty }{B}_{n}{K}_{n}({k}_{2s}r)\text{sin}[n(\theta -{\theta }_{0})]*{e}^{i{k}_{z2s}(z-{z}_{0})}$$

11 $${\psi }_{2}\left(r,\theta ,z\right)=\sum_{n=0}^{\infty }{C}_{n}{K}_{n}({k}_{2s}r)\text{cos}[n(\theta -{\theta }_{0})]*{e}^{i{k}_{z2s}(z-{z}_{0})}$$

where ${A}_{n}$, ${B}_{n}$, ${C}_{n}$ and ${D}_{n}$ are the desired parameters (relative amplitude) and the other variables in Eqs. (8)–(11) have the same meanings as those in Eq. (4). The wave propagation pattern at the interface is shown in Fig. 2, in which we define the plane passing through the source and perpendicular to the z-axis as the xoy plane (z_0_ = 0).

According to Eqs. A4-A6, it can be determined that P and SV are coupled, while SH is independent. Therefore, a P-wave point source will not generate an SH wave. In the xoy plane, the slowness component in the z direction is zero. Substituting this into the displacement-stress formulas in Appendix A, the expressions related to SV are zero. Based on the above content, it can be concluded that in the xoy plane, with the point source placed anywhere, only P-waves propagate. In the subsequent content, we will still present the equilibrium equations considering the existence of SH and SV waves. Readers can verify the correctness of this conclusion through the derived formulas or computer calculation results.

## Mechanism around the hole

According to the Snell’ law, the slowness of the scattered waves along the z direction is equal (i.e. *k*_*zip*_ = *k*_*zis*_ = ${k}_{z}$). The displacement and stress components of the scattered waves (see Appendix A) are substituted into Eq. (1) and obtain the following matrix:

12 $${[{a}_{ij}]}_{4x4}{[{b}_{j}]}_{4x1}={[{c}_{i}]}_{4x1}$$

where $\left[{b}_{j}\right]={[{D}_{n},{A}_{n},{B}_{n},{C}_{n}]}^{T}$ is the relative amplitude, which needs to be solved. $[{a}_{ij}]$ and ${[{c}_{i}]}_{4x1}$ are both given in Appendix B.

The six stress components of the hole model is

13 $${[\sigma ]}_{ij}=\left[\begin{array}{ccc}{\sigma }_{rr}^{(2)}& {\sigma }_{r\theta }^{(2)}& {\sigma }_{rz}^{(2)}\\ {\sigma }_{r\theta }^{(2)}& {\sigma }_{\theta \theta }^{(2)}& {\sigma }_{\theta z}^{(2)}\\ {\sigma }_{rz}^{(2)}& {\sigma }_{\theta z}^{(2)}& {\sigma }_{zz}^{(2)}\end{array}\right]$$

whose elements in Eq. (13) are expressed as with the source in the liquid medium

14a $${\sigma }_{rr}^{(2)}=\{\sum_{n=0}^{\infty }[{a}_{22}{A}_{n}+{a}_{23}{B}_{n}+{a}_{24}{C}_{n}]\text{cos}\left[n\left(\theta -{\theta }_{0}\right)\right]\}{e}^{i{k}_{z}(z-{z}_{0})}$$

14b $${\sigma }_{r\theta }^{(2)}=\{\sum_{n=0}^{\infty }[{a}_{32}{A}_{n}+{a}_{33}{B}_{n}+{a}_{34}{C}_{n}]\text{sin}\left[n\left(\theta -{\theta }_{0}\right)\right]\}{e}^{i{k}_{z}(z-{z}_{0})}$$

14c $${\sigma }_{rz}^{(2)}=\{\sum_{n=0}^{\infty }[{a}_{42}{A}_{n}+{a}_{43}{B}_{n}+{a}_{44}{C}_{n}]\text{cos}\left[n\left(\theta -{\theta }_{0}\right)\right]\}{e}^{i{k}_{z}(z-{z}_{0})}$$

14d $${\sigma }_{\theta \theta }^{(2)}=\{\sum_{n=0}^{\infty }[{d}_{12}{A}_{n}+{d}_{13}{B}_{n}+{d}_{14}{C}_{n}]\text{cos}\left[n\left(\theta -{\theta }_{0}\right)\right]\}{e}^{i{k}_{z}(z-{z}_{0})}$$

14e $${\sigma }_{zz}^{(2)}=\{\sum_{n=0}^{\infty }[{d}_{22}{A}_{n}+{d}_{24}{C}_{n}]\text{cos}\left[n\left(\theta -{\theta }_{0}\right)\right]\}{e}^{i{k}_{z}(z-{z}_{0})}$$

14f $${\sigma }_{\theta z}^{(2)}=\{\sum_{n=0}^{\infty }[{d}_{32}{A}_{n}+{d}_{33}{B}_{n}+{d}_{34}{C}_{n}]\text{sin}\left[n\left(\theta -{\theta }_{0}\right)\right]\}{e}^{i{k}_{z}(z-{z}_{0})}$$

where $[{d}_{ij}]$ is listed in Appendix C. We could solve the characteristic roots of Eq. (13) (i.e. principal stresses ${\sigma }_{i}$) and calculate the equivalent stress (${\sigma }_{0}=\sqrt{\sum_{i=1}^{3}{\sigma }_{i}^{2}}$) under point-source disturbance.

If we move the source into formation and observe the response of hole, the stress components in Eqs. (14a)–(14f) would be changed as

15a $${\sigma }_{rr}^{(2)}=\{\sum_{n=0}^{\infty }[-{c}_{2}+{a}_{22}{A}_{n}+{a}_{23}{B}_{n}+{a}_{24}{C}_{n}]\text{cos}\left[n\left(\theta -{\theta }_{0}\right)\right]\}{e}^{i{k}_{z}(z-{z}_{0})}$$

15b $${\sigma }_{r\theta }^{(2)}=\{\sum_{n=0}^{\infty }[-{c}_{3}+{a}_{32}{A}_{n}+{a}_{33}{B}_{n}+{a}_{34}{C}_{n}]\text{sin}\left[n\left(\theta -{\theta }_{0}\right)\right]\}{e}^{i{k}_{z}(z-{z}_{0})}$$

15c $${\sigma }_{rz}^{(2)}=\{\sum_{n=0}^{\infty }[-{c}_{4}+{a}_{42}{A}_{n}+{a}_{43}{B}_{n}+{a}_{44}{C}_{n}]\text{cos}\left[n\left(\theta -{\theta }_{0}\right)\right]\}{e}^{i{k}_{z}(z-{z}_{0})}$$

15d $${\sigma }_{\theta \theta }^{(2)}=\{\sum_{n=0}^{\infty }[{d}_{11}+{d}_{12}{A}_{n}+{d}_{13}{B}_{n}+{d}_{14}{C}_{n}]\text{cos}\left[n\left(\theta -{\theta }_{0}\right)\right]\}{e}^{i{k}_{z}(z-{z}_{0})}$$

15e $${\sigma }_{zz}^{(2)}=\{\sum_{n=0}^{\infty }[{d}_{21}+{d}_{22}{A}_{n}+{d}_{24}{C}_{n}]\text{cos}\left[n\left(\theta -{\theta }_{0}\right)\right]\}{e}^{i{k}_{z}(z-{z}_{0})}$$

15f $${\sigma }_{\theta z}^{(2)}=\{\sum_{n=0}^{\infty }[{d}_{31}+{d}_{32}{A}_{n}+{d}_{33}{B}_{n}+{d}_{34}{C}_{n}]\text{sin}\left[n\left(\theta -{\theta }_{0}\right)\right]\}{e}^{i{k}_{z}(z-{z}_{0})}$$

## Numerical examples

In the preceding section, we derived the analytical approach to analyze the response mechanism of the hole to the 3D point-source dynamic disturbance. According to the derived approach, we build numerical examples to observe how model parameters affect the stress distribution. For example, based on Table 1, we generated curves of the equivalent stress at the interface of solid and liquid with the source located in the liquid medium.

**Table 1:** Parameters for the hole model.

$\uplambda$ (Gpa)	$\upmu$ (Gpa)	$\uprho$ (g/cm^3^)	Model	${\text{k}}_{z}$	${r}_{a}$ *m*	${r}_{0}$ *m*	$f$ (Hz)
10	0	1	I	$\frac{2\pi f}{100}$	0.1	0.03	1e1, 1e2, 1e3
20	10	1.6	II	$\frac{2\pi f}{10}$	0.1,0.12,0.15	0.5	1e2

**Figure 3:**
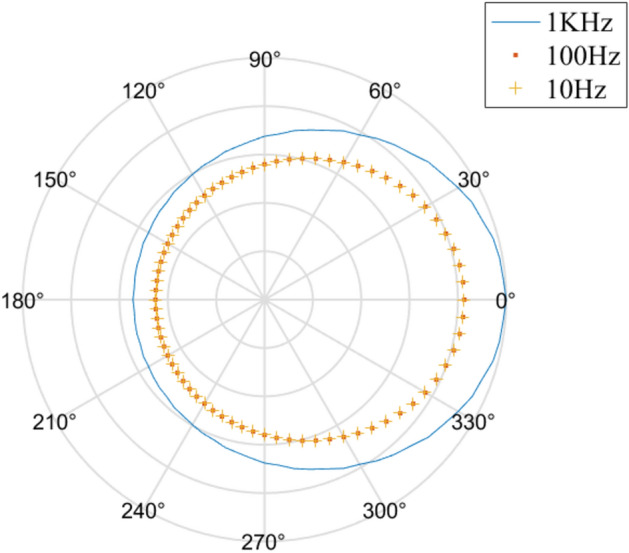
Effects of frequency on the equivalent stress.

Based on Model I from Table 1, Fig. 3 is generated to observe the impact of frequency on the equivalent stress (the results of the calculation are divided by its maximum).

From Fig. 3, we could conclude that as frequency increases, the equivalent stress is becoming larger; the maximum stress occurs at the hole closest to the point source.

**Figure 4:**
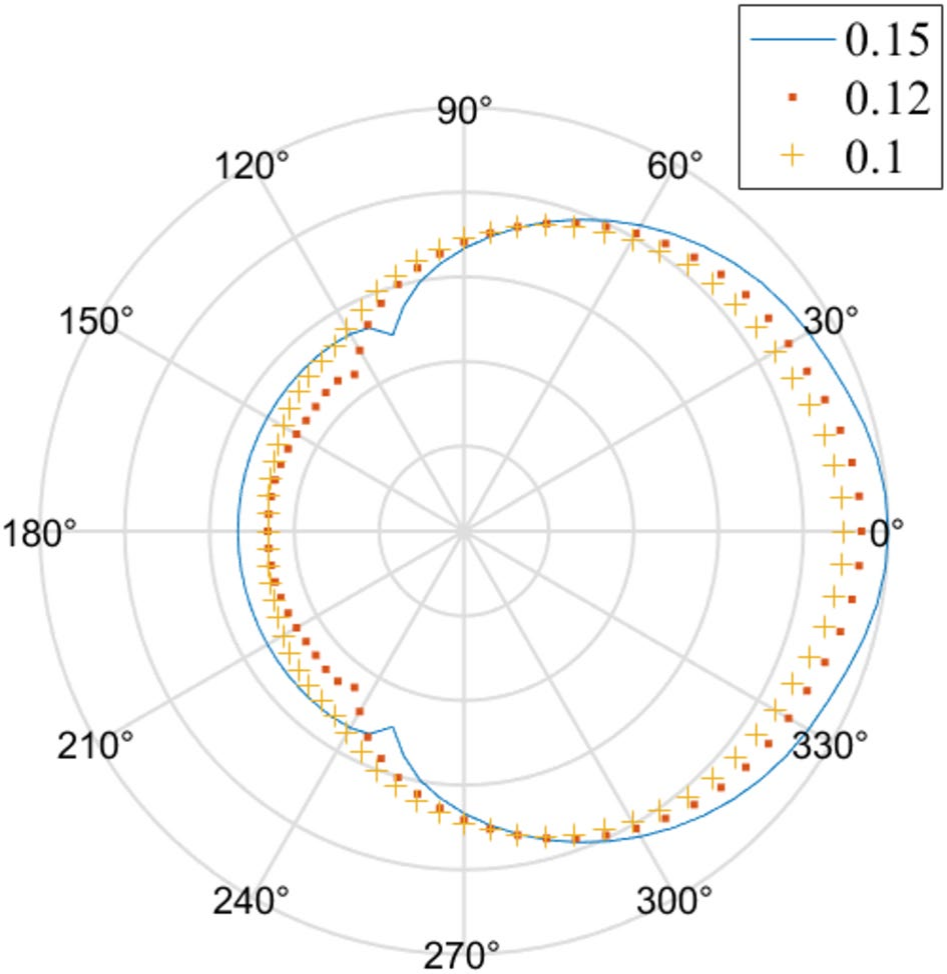
Effects of the hole radius on the equivalent stress.

Based on Model II from Table 1, Fig. 4 is generated to observe the impact of the hole radius on the equivalent stress:

Due to the exponential differences in the calculation results from Model II, take the base-10 logarithm of the results, and then generate Fig. 4 according to the method explained in Fig. 3. From Fig. 4, we can conclude that as the hole radius increases, the corresponding stress magnitude at the same location does not monotonically increase or decrease. This situation generally occurs at locations farther from the point source (e.g. azimuth from 60° to 300°).

We build Model III to study on the damping effect of the hole on dynamic disturbance through the stress ratio of after and before excavation. The parameters for Model III are listed in Table 2.

**Table 2:** Model parameters for model III.

$\uplambda$ (Gpa)	$\upmu$ (Gpa)	$\uprho$ (g/cm^3^)	${\text{k}}_{z}$	${r}_{a}$ *m*	${r}_{0}$ *m*	$f$ (Hz)
10	0	1	$\frac{2\pi f}{100},\frac{2\pi f}{1000}$	0.15	0.5	1e2
20	10	1.6				

**Figure 5:**
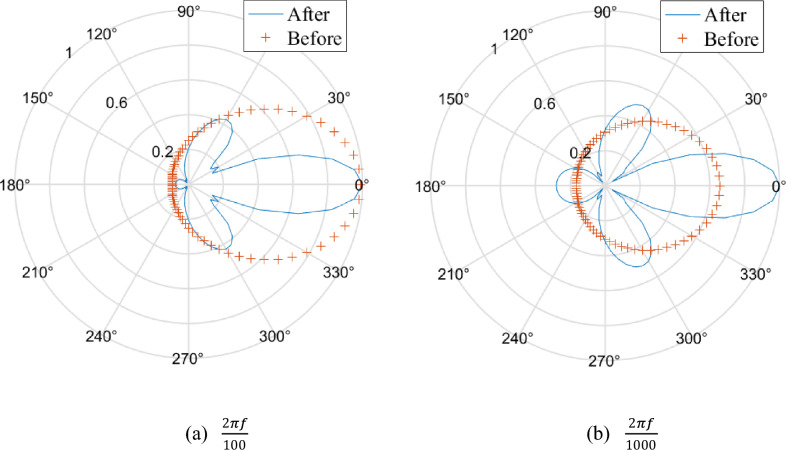
Comparison of the equivalent stresses between after and before excavation.

Based on Model III, we generate Fig. 5 in the same way as Fig. 3 and conclude that when the slowness component along the z direction is becoming smaller, in some parts of the hole, the equivalent stress is amplified rather than reduced. The difference of solving the equivalent stresses after and before excavation is that we substitute *A*_*n*_ = *B*_*n*_ = *C*_*n*_ = 0 into Eqs. (15a)–(15f). The hole may amplify the equivalent stress behind the hole within the plane close to the xoy plane.

**Figure 6:**
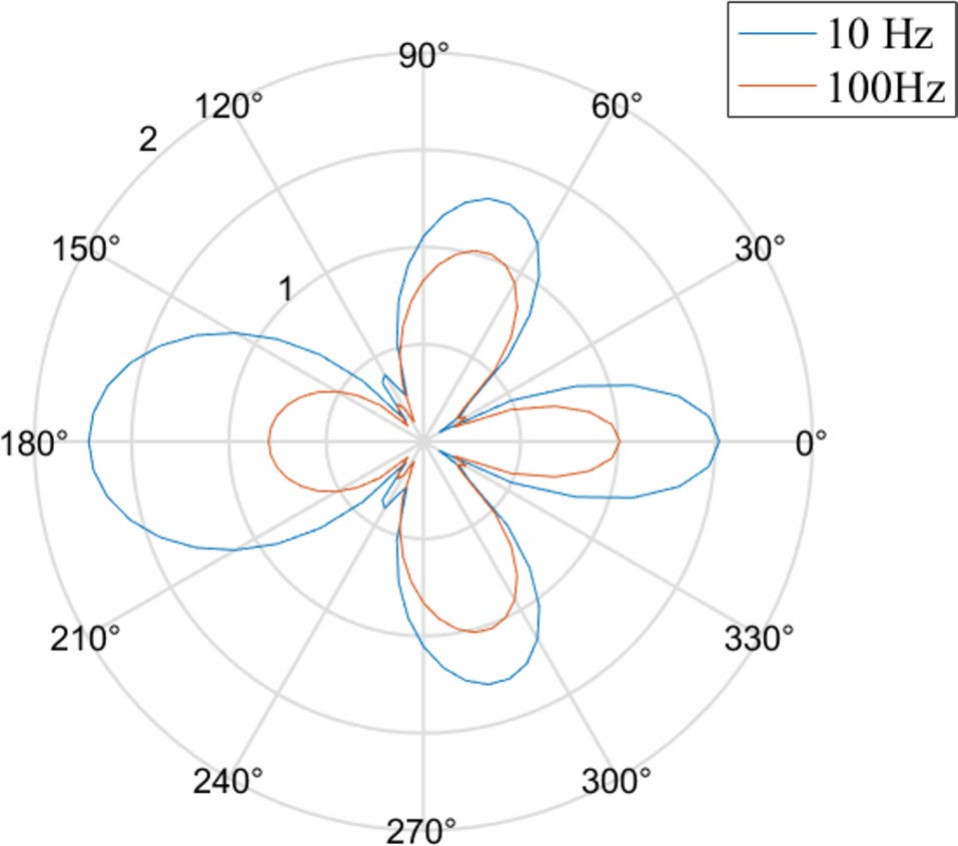
The attenuation effect of the hole on dynamic disturbance.

We calculate the ratio of the equivalent stresses after and before excavation from Fig. 5a at 10 and 100 Hz and generate Fig. 6. From Fig. 6, we discover that the hole attenuates high-frequency disturbances much better than low-frequency disturbances.

## Discussions

In the absence of liquid in the hole, we only change Eq. (12) to the following according to Eq. (2)

16 $$\sum_{j=2}^{4}{a}_{ij}{b}_{j}={c}_{i},i=\text{2,3},4$$

and the remaining process for the equivalent stress without liquid is the same as with liquid. Also the expressions for stress components without liquid are the same as Eqs. (15a)–(15f). In this section, using a point source vibration as an example, we study the impact of ${r}_{0}/{r}_{a}$ on dynamic disturbance.

**Figure 7:**
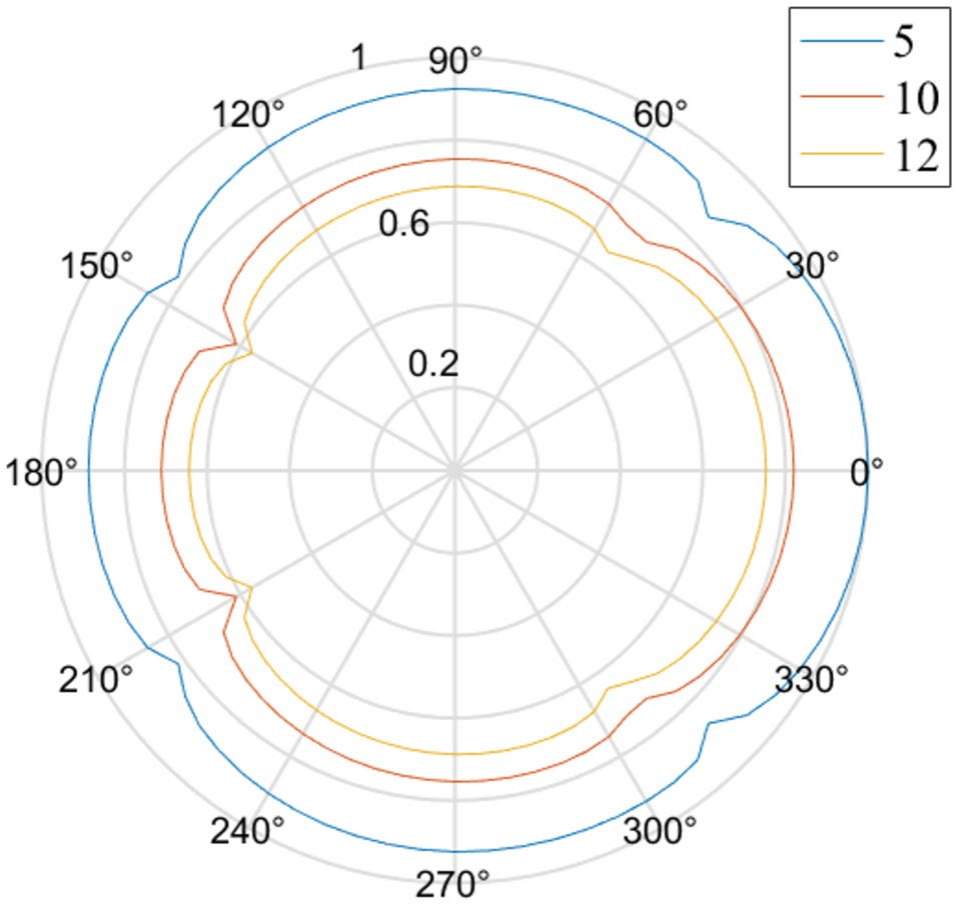
The effect of the source position on dynamic disturbance.

According to Model IV from Table 3, we discuss the effect of the source position on dynamic disturbance and draw Fig. 7, which is generated in the same way as Fig. 4. From Fig. 7, we conclude that the relative stress around the hole is almost unchanged at r_0_/r_a_ > 10.

**Table 3:** Parameters for the effect of the source position on dynamic disturbance.

$\uplambda$ (Gpa)	$\upmu$ (Gpa)	$\uprho$ (g/cm^3^)	Model	${\text{k}}_{z}$	${r}_{a}$(m)	${r}_{0}/{r}_{a}$	$f$ (Hz)
0	0	0	IV	$\frac{2\pi f}{1000}$	1	5, 10, 12	1e2
20	10	1.6

In the above numerical calculations, two main issues need to be addressed: one is the summation of infinite series, and the other is the singularity of matrix coefficients. The issue of summing infinite series can be resolved by terminating the calculation when the ratio of the results of the nth and (n + 1)th iterations reaches a certain threshold. For example, when the value of n is around 30, the calculation result is almost unchanged as n increases. As for the singularity of [*a*_*ij*_] from Eq. (12), normalize the coefficients of each row of the matrix. If there are large differences within the same row, adjust the parameter units, such as changing from GPa to MPa or Pa, and try different ways to reduce the magnitude differences between the coefficients.

## Conclusions

According to the boundary conditions, we derive the analytical solution to solve the equivalent stress distribution around the hole, which considers the position of the 3D point source. We build numerical examples to observe the influence of model parameters on the equivalent stress of the hole and can reach the following conclusions:

1. In the plane that contains the source and perpendicular to the hole axis, it won’t generate the shear waves. Out of the plane, only slow shear (SV) wave is generated.
2. As frequency increases, the equivalent stress around the borehole is becoming larger. However, at a constant frequency, the stress far from the point source may be larger than that close to the point source within the plane close to the xoy plane.
3. Comparing the equivalent stress states behind the hole before and after excavation, the borehole has a good attenuation on high-frequency disturbances, but it can amplify low-frequency disturbances.
4. As the distance between the source and the borehole axis increases (usually over 10 r_0_), the relative equivalent stress distribution at the hole is almost unchanged at a constant *k*_*z*_.

### Supplementary Information

Supplementary Information.

## Data Availability

The data that supports the findings of this study is available from the corresponding author upon reasonable request.
