# Supplementary material for: Dynamic response mechanism of the hole subjected to the 3D point source disturbance in an isotropic formation
Source: Sci Rep. 2024 Jul 19;14:16716. doi: 10.1038/s41598-024-67417-8 (PMC11271553; doi:10.1038/s41598-024-67417-8)
Supplement: Supplementary file 1 — Supplementary Information. [file 41598_2024_67417_MOESM1_ESM.docx]

**Appendix A: Frequency Equation**

The motion equations for an isotropic elastic medium are changed, in invariant form ^[36]^,

$\mu\Delta\boldsymbol{u}+\left( \lambda+\mu\right)\nabla\nabla\cdot\boldsymbol{u}=\rho\frac{\partial^{2}\boldsymbol{u}}{\partial t^{2}}$ (A1)

where $\boldsymbol{u}$ is the displacement fields, $\left( \lambda,\mu\right)$ are the Lame’s constants, $\Delta$ is the 3D Laplace operator, and $\rho$ is the density. Based on the Helmholtz theorem to decompose ^[6,37]^:

$\boldsymbol{u=}\nabla\phi+\nabla\times(\chi\hat{e}_{z})+\nabla\times\nabla\times(\psi\hat{e}_{z})$ (A3)

and simplify Eq.A1 to obtain the following functions

$\left( \Delta-\frac{\partial^{2}}{\partial z^{2}} \right)\left( \left( \lambda+2\mu\right)\Delta-\rho\frac{\partial^{2}}{\partial t^{2}} \right)\phi+\frac{\partial}{\partial z}\left( \mu\Delta-\rho\frac{\partial^{2}}{\partial t^{2}} \right)\psi=0$ (A4)

$\frac{\partial}{\partial z}\left( \left( \lambda+2\mu\right)\Delta-\rho\frac{\partial^{2}}{\partial t^{2}} \right)\phi+\left( \Delta-\frac{\partial^{2}}{\partial z^{2}} \right)\left( \mu\Delta-\rho\frac{\partial^{2}}{\partial t^{2}} \right)\psi=0$ (A5)

$\left( \Delta-\frac{\partial^{2}}{\partial z^{2}} \right)\left( \mu\Delta-\rho\frac{\partial^{2}}{\partial t^{2}} \right)\chi=0$ (A6)

where $\phi$, $\psi$, and $\chi$ represent *P*, *SV* and *SH* waves, respectively, and $\hat{e}_{z}$ is the z-axis unit vector. Moreover, Eq. A3 can be furtherly expanded as

$u_{r}=\frac{\partial\phi}{\partial r}+\frac{\partial\chi}{r\partial\theta}+\frac{\partial^{2}\psi}{\partial r\partial z}$ (A7)

$u_{\theta}=\frac{\partial\phi}{r\partial\theta}-\frac{\partial\chi}{\partial r}+\frac{1}{r}\frac{\partial^{2}\psi}{\partial\theta\partial z}$ (A8)

$u_{z}=\frac{\partial\phi}{\partial z}-(\frac{\partial^{2}}{\partial r^{2}}+\frac{\partial}{r\partial r}+\frac{\partial^{2}}{r^{2}{\partial\theta}^{2}})\psi$ (A9)

The displacement-strain relation ^[8]^

$\left\{ \begin{matrix} \varepsilon_{rr}=\frac{\partial u_{r}}{\partial r} \\ \varepsilon_{\theta\theta}=\frac{\partial u_{\theta}}{r\partial\theta}+\frac{u_{r}}{r} \\ \varepsilon_{r\theta}=\frac{\partial u_{r}}{r\partial\theta}+\frac{\partial u_{\theta}}{\partial r}-\frac{u_{\theta}}{r} \\ \varepsilon_{rz}=\frac{\partial u_{r}}{\partial z}+\frac{\partial u_{z}}{\partial r} \\ \varepsilon_{\theta z}=\frac{\partial u_{z}}{r\partial\theta}+\frac{\partial u_{\theta}}{\partial z} \\ \varepsilon_{zz}=\frac{\partial u_{z}}{\partial z} \end{matrix} \right.$ (A10)

The strain-stress relation

$\left\{ \begin{matrix} \sigma_{rr}\boldsymbol{=}\left( \lambda+2\mu\right)\varepsilon_{rr}+\lambda\left( \varepsilon_{zz}+\varepsilon_{\theta\theta} \right) \\ \sigma_{\theta\theta}\boldsymbol{=}\left( \lambda+2\mu\right)\varepsilon_{\theta\theta}+\lambda\left( \varepsilon_{zz}+\varepsilon_{rr} \right) \\ \sigma_{zz}\boldsymbol{=}\left( \lambda+2\mu\right)\varepsilon_{zz}+\lambda\left( \varepsilon_{rr}+\varepsilon_{\theta\theta} \right) \\ \sigma_{r\theta}\boldsymbol{=}\mu\varepsilon_{r\theta} \\ \sigma_{rz}\boldsymbol{=}\mu\varepsilon_{rz} \\ \sigma_{\theta z}\boldsymbol{=}\mu\varepsilon_{\theta z} \end{matrix} \right.$ (A11)

which are expanded as

$\sigma_{rr}\boldsymbol{=}\left\{ \begin{matrix} \left( \lambda+2\mu\right)\left( \frac{\partial^{2}\phi}{\partial r^{2}}+\frac{\partial^{2}\chi}{r\partial r\partial\theta}-\frac{1}{r^{2}}\frac{\partial\chi}{\partial\theta}+\frac{\partial^{3}\psi}{\partial r^{2}\partial z} \right) \\ +\lambda\left[ \frac{\partial^{2}\phi}{\partial z^{2}}-\left( \frac{\partial^{3}}{\partial r^{2}\partial z}+\frac{\partial^{2}}{r\partial r\partial z}+\frac{\partial^{3}}{r^{2}{\partial\theta}^{2}\partial z} \right)\psi\right] \\ +\frac{\lambda}{r}(\frac{\partial\phi}{\partial r}+\frac{\partial\chi}{r\partial\theta}+\frac{\partial^{2}\psi}{\partial r\partial z}+\frac{\partial^{2}\phi}{r{\partial\theta}^{2}}-\frac{\partial^{2}\chi}{\partial\theta\partial r}+\frac{1}{r}\frac{\partial^{3}\psi}{{\partial\theta}^{2}\partial z}) \end{matrix} \right.$ (A12)

$\sigma_{\theta\theta}\boldsymbol{=}\left\{ \begin{matrix} \lambda\left( \frac{\partial^{2}\phi}{\partial r^{2}}+\frac{\partial^{2}\chi}{r\partial r\partial\theta}-\frac{1}{r^{2}}\frac{\partial\chi}{\partial\theta}+\frac{\partial^{3}\psi}{\partial r^{2}\partial z} \right) \\ +\lambda\left[ \frac{\partial^{2}\phi}{\partial z^{2}}-\left( \frac{\partial^{3}}{\partial r^{2}\partial z}+\frac{\partial^{2}}{r\partial r\partial z}+\frac{\partial^{3}}{r^{2}{\partial\theta}^{2}\partial z} \right)\psi\right] \\ +\frac{\left( \lambda+2\mu\right)}{r}(\frac{\partial\phi}{\partial r}+\frac{\partial\chi}{r\partial\theta}+\frac{\partial^{2}\psi}{\partial r\partial z}+\frac{\partial^{2}\phi}{r{\partial\theta}^{2}}-\frac{\partial^{2}\chi}{\partial\theta\partial r}+\frac{1}{r}\frac{\partial^{3}\psi}{{\partial\theta}^{2}\partial z}) \end{matrix} \right.$ (A13)

$\sigma_{zz}\boldsymbol{=}\left\{ \begin{matrix} \lambda\left( \frac{\partial^{2}\phi}{\partial r^{2}}+\frac{\partial^{2}\chi}{r\partial r\partial\theta}-\frac{1}{r^{2}}\frac{\partial\chi}{\partial\theta}+\frac{\partial^{3}\psi}{\partial r^{2}\partial z} \right) \\ +\left( \lambda+2\mu\right)\left[ \frac{\partial^{2}\phi}{\partial z^{2}}-\left( \frac{\partial^{3}}{\partial r^{2}\partial z}+\frac{\partial^{2}}{r\partial r\partial z}+\frac{\partial^{3}}{r^{2}{\partial\theta}^{2}\partial z} \right)\psi\right] \\ +\frac{\lambda}{r}(\frac{\partial\phi}{\partial r}+\frac{\partial\chi}{r\partial\theta}+\frac{\partial^{2}\psi}{\partial r\partial z}+\frac{\partial^{2}\phi}{r{\partial\theta}^{2}}-\frac{\partial^{2}\chi}{\partial\theta\partial r}+\frac{1}{r}\frac{\partial^{3}\psi}{{\partial\theta}^{2}\partial z}) \end{matrix} \right.$ (A14)

$\sigma_{r\theta}\boldsymbol{=}\left\{ \begin{matrix} \frac{\mu}{r}(-\frac{2\partial\phi}{r\partial\theta}+2\frac{\partial^{2}\phi}{\partial r\partial\theta}) \\ +\frac{\mu}{r}(-r\frac{\partial^{2}\chi}{\partial r^{2}}+\frac{\partial\chi}{\partial r}+\frac{\partial^{2}\chi}{r{\partial\theta}^{2}}) \\ +2\frac{\mu}{r}(-\frac{1}{r}\frac{\partial^{2}\psi}{\partial\theta\partial z}+\frac{\partial^{3}\psi}{\partial r\partial\theta\partial z}) \end{matrix} \right.$ (A15)

$\sigma_{rz}\boldsymbol{=}\left\{ \begin{matrix} 2\mu\frac{\partial^{2}\phi}{\partial r\partial z} \\ +\frac{\mu}{r}\frac{\partial^{2}\chi}{\partial\theta\partial z} \\ -\mu(\frac{\partial^{3}}{\partial r^{3}}+\frac{1}{r}\frac{\partial^{2}}{\partial r^{2}}-\frac{1}{r^{2}}\frac{\partial}{\partial r}+\frac{1}{r^{2}}\frac{\partial^{3}}{{\partial\theta}^{2}\partial r}-\frac{2}{r^{3}}\frac{\partial^{2}}{{\partial\theta}^{2}}-\frac{\partial^{3}}{{\partial z}^{2}\partial r})\psi\end{matrix} \right.$ (A16)

$\sigma_{\theta z}\boldsymbol{=}\left\{ \begin{matrix} \frac{2\mu}{r}\frac{\partial^{2}\phi}{\partial z\partial\theta} \\ -\mu\frac{\partial^{2}\chi}{\partial r\partial z} \\ -\frac{\mu}{r}\left( \frac{\partial^{3}}{\partial\theta\partial r^{2}}+\frac{\partial^{2}}{r\partial r\partial\theta}+\frac{\partial^{3}}{r^{2}{\partial\theta}^{3}}-\frac{\partial^{3}}{\partial z^{2}\partial\theta} \right)\psi\end{matrix} \right.$ (A17)

The above displacement and stress components are used to build boundary equations and then discuss the crack initiation due to the stress concentration around the borehole. Moreover, Eqs. A4-A6 show that P- and SV-waves are coupled and if assuming kz=0, we discover that SV and SH waves won’t be generated at the xoy plane based on Eqs. 12-17.

**Appendix B: Elements of the Matrices for the borehole model**

The elements in Eq. 12 are listed as

| $a_{11}=-[\frac{n}{r_{a}}I_{n}\left( k_{1p}r_{a} \right)+k_{1p}I_{n+1}\left( k_{1p}r_{a} \right)]$ | $a_{12}=\frac{n}{r_{a}}K_{n}\left( k_{2p}r_{a} \right)-k_{2p}K_{n+1}\left( k_{2p}r_{a} \right)$ |
| --- | --- |
| $a_{13}=\frac{n}{r_{a}}K_{n}\left( k_{2s}r_{a} \right)$ | $a_{14}=ik_{z}[\frac{n}{r_{a}}K_{n}\left( k_{2s}r_{a} \right)-k_{2s}K_{n+1}\left( k_{2s}r_{a} \right)]$ |
| $a_{21}=\omega^{2}\rho_{1}I_{n}\left( k_{1p}r_{a} \right)$ | $a_{22}=\mu_{2}\left\{ \left[ \frac{2n\left( n-1 \right)}{r_{a}^{2}}+2k_{2p}^{2} \right]K_{n}\left( k_{2p}r_{a} \right)+2\frac{k_{2p}}{r_{a}}K_{n+1}\left( k_{2p}r_{a} \right) \right\}+\lambda_{2}K_{n}\left( k_{2p}r_{a} \right)(k_{2p}^{2}-k_{z}^{2})$ |
| $a_{23}=2\mu_{2}\frac{n}{r_{a}}[\frac{n-1}{r_{a}}K_{n}\left( k_{2s}r_{a} \right)-k_{2s}K_{n+1}\left( k_{2s}r_{a} \right)]$ | $a_{24}=2ik_{z}\mu_{2}\{\left[ \frac{n\left( n-1 \right)}{r_{a}^{2}}+k_{2s}^{2} \right]K_{n}\left( k_{2s}r_{a} \right)+\frac{k_{2s}}{r_{a}}K_{n+1}\left( k_{2s}r_{a} \right)\}$ |
| $a_{32}=-2\mu_{2}\frac{n}{r_{a}}[\frac{n-1}{r_{a}}K_{n}\left( k_{2p}r_{a} \right)-k_{2p}K_{n+1}\left( k_{2p}r_{a} \right)]$ | $a_{33}=-\mu_{2}\{\left[ \frac{2n\left( n-1 \right)}{r_{a}^{2}}+k_{2s}^{2} \right]K_{n}\left( k_{2s}r_{a} \right)+\frac{2k_{2s}}{r_{a}}K_{n+1}\left( k_{2s}r_{a} \right)\}$ |
| $a_{34}=2ik_{z}\mu_{2}\frac{n}{r_{a}}[\frac{1-n}{r_{a}}K_{n}\left( k_{2s}r_{a} \right)+k_{2s}K_{n+1}\left( k_{2s}r_{a} \right)]$ | $a_{42}=2ik_{z}\mu_{2}[\frac{n}{r_{a}}K_{n}\left( k_{2p}r_{a} \right)-k_{2p}K_{n+1}\left( k_{2p}r_{a} \right)]$ |
| $a_{43}=ik_{z}\mu_{2}\frac{n}{r_{a}}K_{n}\left( k_{2s}r_{a} \right)$ | $a_{44}=\mu_{2}\left( k_{s}^{2}-2k_{z}^{2} \right)[\frac{n}{r_{a}}K_{n}\left( k_{2s}r_{a} \right)-k_{2s}K_{n+1}\left( k_{2s}r_{a} \right)]$ |

Elements not listed are zero and *k_s_* is the SH (or SV) wavenumber. If the source is located in Medium 1 and Medium 2, respectively, the expression of $[c_{i}]$ is

$\frac{{[c_{i}]}_{4x1}}{\varepsilon_{n}I_{n}\left( k_{1p}r_{0} \right)}=\left[ \begin{matrix} \frac{n}{r_{a}}K_{n}\left( k_{1p}r_{a} \right)-k_{1p}K_{n+1}\left( k_{1p}r_{a} \right) \\ -\omega^{2}\rho_{1}K_{n}\left( k_{1p}r_{a} \right) \\ 0 \\ 0 \end{matrix} \right]$ (B1)

and

$\frac{-{[c_{i}]}_{4x1}}{\varepsilon_{n}K_{n}\left( k_{2p}r_{0} \right)}=\left[ \begin{matrix} \frac{n}{r_{a}}I_{n}\left( k_{2p}r_{a} \right)+k_{2p}I_{n+1}\left( k_{2p}r_{a} \right) \\ \lambda_{2}I_{n}\left( k_{2p}r_{a} \right)\left( k_{2p}^{2}-k_{z}^{2} \right)-2\mu_{2}[\left( \frac{n\left( n-1 \right)}{r_{a}^{2}}+k_{z}^{2} \right)I_{n}\left( k_{2p}r_{a} \right)-\frac{k_{2p}}{r_{a}}I_{n+1}\left( k_{2p}r_{a} \right)] \\ -2\mu_{2}\frac{n}{r_{a}}[\left( \frac{n-1}{r_{a}} \right)I_{n}\left( k_{2p}r_{a} \right)+k_{2p}I_{n+1}\left( k_{2p}r_{a} \right)] \\ 2i\mu_{2}(\frac{n}{r_{a}}I_{n}\left( k_{2p}r_{a} \right)+k_{z}I_{n+1}\left( k_{2p}r_{a} \right)) \end{matrix} \right]$(B2)

**Appendix C Elements in Eq.14**

| $\frac{d_{11}}{\varepsilon_{n}K_{n}\left( k_{2p}r_{0} \right)}=I_{n}\left( k_{2p}r_{a} \right)[\lambda_{2}(k_{2p}^{2}-k_{z}^{2})-\frac{2\mu_{2}n\left( n-1 \right)}{r_{a}^{2}}]+2\mu_{2}\frac{k_{2p}}{r_{a}}I_{n+1}\left( k_{2p}r_{a} \right)$ | $d_{12}=-\mu_{2}\left\{ \frac{2n\left( n-1 \right)}{r_{a}^{2}}K_{n}\left( k_{2p}r_{a} \right)+2\frac{k_{2p}}{r_{a}}K_{n+1}\left( k_{2p}r_{a} \right) \right\}+\lambda_{2}K_{n}\left( k_{2p}r_{a} \right)(k_{2p}^{2}-k_{z}^{2})$ |
| --- | --- |
| $d_{13}=-a_{23}$ | $d_{14}=-2ik_{z}\mu_{2}[\frac{n\left( n-1 \right)}{r_{a}^{2}}K_{n}\left( k_{2s}r_{a} \right)+\frac{k_{2s}}{r_{a}}K_{n+1}\left( k_{2s}r_{a} \right)]$ |
| $\frac{d_{21}}{\varepsilon_{n}K_{n}\left( k_{2p}r_{0} \right)}=-I_{n}\left( k_{2p}r_{a} \right)[\left( \lambda_{2}+2\mu_{2} \right)k_{z}^{2}-\lambda_{2}k_{2p}^{2}]$ | $d_{22}=-K_{n}\left( k_{2p}r_{a} \right)[\left( \lambda_{2}+2\mu_{2} \right)k_{z}^{2}-\lambda_{2}k_{2p}^{2}]$ |
| $d_{24}=-2ik_{z}\mu_{2}k_{2s}^{2}K_{n}\left( k_{2s}r_{a} \right)$ | $\frac{d_{31}}{\varepsilon_{n}K_{n}\left( k_{2p}r_{0} \right)}=-2ik_{z}\mu_{2}\frac{n}{r_{a}}I_{n}\left( k_{2p}r_{a} \right)$ |
| $d_{32}=-2ik_{z}\mu_{2}\frac{n}{r_{a}}K_{n}\left( k_{2p}r_{a} \right)$ | $d_{33}=ik_{z}\mu_{2}[k_{2s}K_{n+1}\left( k_{2s}r_{a} \right)-\frac{n}{r_{a}}K_{n}\left( k_{2s}r_{a} \right)]$ |
| $d_{34}=\mu_{2}\frac{n}{r_{a}}K_{n}\left( k_{2s}r_{a} \right)(k_{2s}^{2}+k_{z}^{2})$ |  |
